# Simultaneous Emerging Contaminant Removal and H_2_O_2_ Generation Through Electron Transfer Carrier Effect of Bi─O─Ce Bond Bridge Without External Energy Consumption

**DOI:** 10.1002/advs.202308519

**Published:** 2024-06-03

**Authors:** Yingtao Sun, Xuanying Cai, Yufeng Lai, Chun Hu, Lai Lyu

**Affiliations:** ^1^ Key Laboratory for Water Quality and Conservation of the Pearl River Delta Ministry of Education Institute of Environmental Research at Greater Bay Guangzhou University Guangzhou 510006 China; ^2^ Institute of Rural Revitalization Guangzhou University Guangzhou 510006 China

**Keywords:** dual reaction centers, H_2_O_2_ generation, oxygen activation, zero external energy consumption

## Abstract

Conventional advanced oxidation processes (AOPs) require significant external energy consumption to eliminate emerging contaminants (ECs) with stable structures. Herein, a catalyst consisting of nanocube BiCeO particles (BCO‐NCs) prepared by an impregnation‐hydrothermal process is reported for the first time, which is used for removing ECs without light/electricity or any other external energy input in water and simultaneous in situ generation of H_2_O_2_. A series of characterizations and experiments reveal that dual reaction centers (DRC) which are similar to the valence band/conducting band structure are formed on the surface of BCO‐NCs. Under natural conditions without any external energy consumption, the BCO‐NCs self‐purification system can remove more than 80% of ECs within 30 min, and complete removal of ECs within 30 min in the presence of abundant electron acceptors, the corresponding second‐order kinetic constant is increased to 3.62 times. It is found that O_2_ can capture electrons from ECs through the Bi─O─Ce bond bridge during the reaction process, leading to the in situ production of H_2_O_2_. This work will be a key advance in reducing energy consumption for deep wastewater treatment and generating important chemical raw materials.

## Graphical abstract

A novel dual reaction centers catalyst consisting of nanocube BiCeO particles (BCO‐NCs) prepared by an impregnation‐hydrothermal process is used for removing ECs without light/electricity or any other external energy input in water and simultaneous in situ generation of H_2_O_2_.

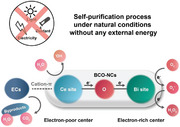

## 1 Introduction

The clustering of industrial areas and population growth in urban areas inevitably generates increasing volumes of wastewater.^[^ ^1^ ^]^ Industrial wastewater contains various pharmaceuticals, pesticides, endocrine disruptors, polycyclic aromatic hydrocarbons, and other emerging contaminants that are ubiquitous in the global water environment. Despite their wide range of uses, the proliferation in the aqueous environment could have potentially adverse effects on humans and other animals.^[^ ^2^ ^]^ In particular, the spread of antibiotics in the environment has become a pressing public health issue. Conventional biological treatment is ineffective for their removal due to the specific nature of antibiotics, which results in frequent detection of antibiotics in the secondary effluent of wastewater treatment plants.^[^ ^3^ ^]^ Therefore, it is important to develop cost‐effective water purification technologies.

To date, advanced oxidation processes (AOPs) have been widely used to treat bio‐refractory organic pollutants in industrial wastewater due to the generation of reactive oxygen species (ROS) to oxidize organic compounds.^[^ ^4^, ^5^ ^]^ To generate large amounts of ROS, exogenous resources and energy are often incorporated into the reaction system, deriving processes such as Fenton/Fenton‐like catalytic oxidation,^[^ ^6^, ^7^ ^]^ photocatalytic oxidation,^[^ ^8^, ^9^, ^10^ ^]^ ozone oxidation,^[^ ^11^, ^12^ ^]^ persulfate catalytic oxidation,^[^ ^13^, ^14^ ^]^ and electrocatalytic oxidation,^[^ ^15^, ^16^, ^17^ ^]^ which lead to high energy consumption of the water purification process. For example, as one of the most widely applied AOPs technologies, conventional homogeneous Fenton reaction has bottlenecks such as inability to separate active components, narrow working pH range (2–4), high H_2_O_2_ consumption and generation of iron sludge.^[^ ^18^, ^19^, ^20^ ^]^ Even the emerging heterogeneous Fenton reaction in recent years also suffers from poor reaction activity and high H_2_O_2_ consumption. The above problems are essential reasons for the high energy consumption of water treatment technology, which is difficult to solve by conventional methods.

Our previous research^[^ ^21^, ^22^, ^23^, ^24^, ^25^, ^26^, ^27^, ^28^, ^29^ ^]^ revealed that the construction of dual reaction centers (DRC) with electron‐directed distribution on the catalyst surface by lattice doping methods is essential to reduce the dependence on H_2_O_2_ and overcome the limitations of the Fenton reaction, which provides inspiration for an in‐depth exploration of tuning the electron distribution on the catalyst surface to completely get rid of the dependence on extra added H_2_O_2_. Bismuth oxide (Bi_2_O_3_), as a member of bismuth‐based semiconductors, is favored for photocatalysis due to its high electrochemical stability, small bandgap (2.6–2.8 eV), and high redox reversibility.^[^ ^30^ ^]^ It is reported^[^ ^31^, ^32^ ^]^ that the photocatalytic performance of Bi oxide nanosheets could be improved by tuning the charge distribution and optical absorption. Apparently, this is achieved by controlling the fine surface structure of the catalyst, indicating that Bi oxides have the advantage of easy surface tuning, which convinced us that Bi oxides have great potential to break the bottleneck of classical Fenton's over‐dependence on H_2_O_2_ via tuning the surface electron distribution to build DRCs. In addition, it is known that photocatalytic process requires UV/visible light to irradiate the semiconductor catalyst, and the electrons in the valence band (VB) are excited and separated from the holes and transferred to the conduction band (CB) to trigger the electron flow for pollutant removal.^[^ ^33^, ^34^ ^]^ However, both photocatalytic and Fenton processes are accompanied by energy consumption of additional H_2_O_2_ or light energy. Therefore, it is necessary to utilize the characteristic advantages of Bi_2_O_3_ to further accurately capture the energy and electrons of endogenous substances in water and construct dual reaction centers (DRCs) with electron‐rich/poor microregion characteristics via surface modulation strategies, which is an innovative and critical strategy to break through the energy consumption problem of water treatment, even though it is a current global scientific challenge.

Herein, in situ surface modulation of classical Bi oxides is performed to prepare DRC catalysts BiCeO‐nanocubes (BCO‐NCs) with VB‐CB‐like structure on the surface by impregnation‐hydrothermal method for the water self‐purification process without any exogenous energy consumption. Structural characterization shows that the electrons on the surface of BCO‐NCs are transferred to Bi centers through Bi─O─Ce bond bridges, resulting in the formation of electron‐rich Bi centers and electron‐poor Ce centers. Typical antibiotic ciprofloxacin is selected as the target contaminant to evaluate BCO‐NCs self‐purification performance under natural conditions without light or any external energy consumption. Surprisingly, H_2_O_2_ is in situ generated in BCO‐NCs self‐purification system simultaneously with water purification. The mechanism of cationic‐π for the cleavage of adsorbed pollutants and the H_2_O_2_ generation from the reduction of O_2_ is proposed based on the experimental results and characterization.

## 2 Results and Discussion

### 2.1 Structural Characterization of BCO‐NCs

**Figure 1:**
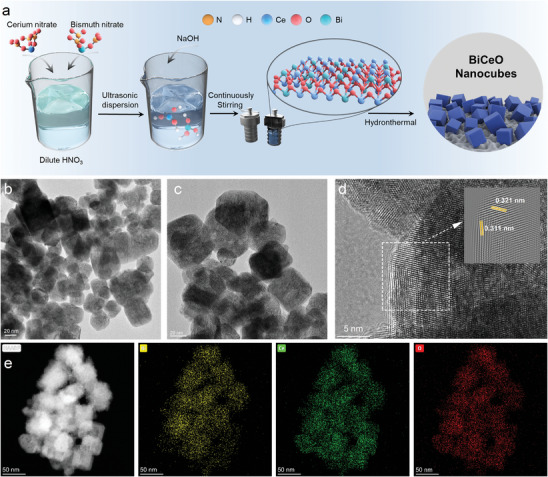
a) Schematic illustration of the preparation process of BCO‐NCs. b,c) TEM image at different scales, d) HRTEM image (the inset shows the inverse Fast Fourier Transformation of TEM), and e) TEM elemental mapping of BCO‐NCs.

The catalyst was prepared by a typical hydrothermal method (**Figure** **1**a). First, Ce(NO_3_)_3·_6H_2_O and Bi(NO_3_)_3·_5H_2_O were added to the nitric acid solution with continuous stirring to form a homogeneous mixed solution of Ce and Bi. Then, NaOH was added and stirred until a stable complex was formed. Finally, the complexes were transferred to an autoclave for hydrothermal reaction, the metal species were embedded with each other in the hydrothermal environment and eventually formed BCO‐NCs.

Scanning electron microscope (SEM) image shows (Figure 1b) that BCO‐NCs consist of uniform cubes with a side length of ≈40 nm and a volume of ≈64 000 nm^3^. Similarly, Transmission electron microscope (TEM) image reveals (Figure 1c) the typical cubic structural features of BCO‐NCs. Two different orientations and sizes of lattice stripes were observed clearly in the High‐resolution transmission electron microscopy (HRTEM) images (Figure 1d), corresponding to the lattice structures of Bi_2_O_3_ and CeO_2_, respectively, indicating that the metal species occurred in mutual lattice substitution rather than simple connecting. This was confirmed by the XRD pattern, as shown in Figure S1 (Supporting Information), CeO pattern corresponds to the cubic crystalline CeO_2_ (PDF#65‐2975), BiO pattern corresponds to the monoclinic crystalline Bi_2_O_3_ (PDF#72‐0398), and the XRD spectrum of BCO‐NCs has all the characteristic peaks of the above samples simultaneously. In addition, the main characteristic peaks of BCO‐NCs include 27.476°, 33.155°, 46.493°, 28.549°, 33.083°, 47.486°, 56.346°, etc., corresponding to (121) (lattice spacing of 0.321 nm), (122), (223) crystallographic planes of Bi_2_O_3_, and (111) (lattice spacing of 0.311 nm), (200), (220), (311) crystal planes of CeO_2_. It is worth noting that the electronegativity of Bi is higher than that of Ce, which allows Bi to show a lower valence state due to the enrichment of electrons around it during the catalyst preparation, while Ce shows a higher valence state due to the loss of electrons, indicating that the BCO‐NCs may construct electron‐poor/rich microregions. TEM elemental mapping was used to analyze the distribution of various elements in BCO‐NCs, as shown in Figure 1e, all elements of nanocubes are uniformly distributed without clusters of metallic species, further confirming that mutual lattice substitution occurs between metallic species. Uniform mutual lattice substitution leads to a specific and stable catalyst structure, providing the potential for efficient removal of ECs from water.

### 2.2 Self‐Purification of Wastewater with Zero External Energy Consumption

As a typical antibiotic ECs, CIP is widely used to treat microbial infections in humans and animals resulting in its consistently high concentration levels in the natural environment, especially in water environment, which could lead to the development of resistance of pathogens at a fast rate and a great threat to human health. Therefore, CIP was chosen as a target ECs to evaluate the wastewater self‐purification performance of the BCO‐NCs system. It is noteworthy that all reactions were performed under mild natural conditions without any external energy or oxidant input.

**Figure 2:**
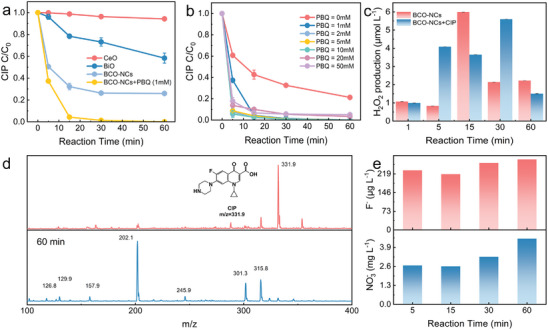
a) Performance of different catalyst suspensions for CIP removal. b) Effect of different PBQ concentrations on CIP removal performance. c) H_2_O_2_ production curves in different suspensions. d) HPLC‐MS spectrum of CIP degradation in the BCO‐NCs system. e) Changes in F^−^ and NO_3_^−^ concentrations in the air‐saturated BCO‐NCs aqueous suspension with initial concentrations of 100 mg L^−1^ CIP.

The BCO‐NCs self‐purification system removed 80% of CIP within 30 min, which was 3.6 and 31.4 times higher compared to the BiO and CeO system (Figure S2, Supporting Information), respectively, which may be related to the structure of bimetallic mutual lattice substitution. It is reported that PBQ has oxidizing properties and could act as an electron acceptor in the reaction process.^[^ ^35^, ^36^ ^]^ Accordingly, a self‐purification process incorporating PBQ was designed (**Figure** **2**a). The removal rate of CIP was significantly increased after adding PBQ to the system, with complete removal within 30 min, the corresponding second‐order kinetic constant was increased by 3.62 times (Figure S2, Supporting Information). This indicates that a strong interfacial electron transfer was present in the self‐purification process of the BCO‐NCs system, which could transfer the electrons inherent of CIP to dissolved oxygen (DO). In addition, the introduction of PBQ accelerated this electron transfer process, leading to a great enhancement of CIP degradation efficiency. To further verify this phenomenon, experiments were set up to investigate the effect of different concentrations of PBQ on the catalytic performance. As expected, the removal of CIP by the BCO‐NCs self‐purification system was significantly enhanced after adding different concentrations of PBQ (Figure 2b). However, the promotion effect for CIP removal efficiency was weakened (Figure S3, Supporting Information) as the concentration of PBQ exceeded 10 mm (20, 50 mm), which might be due to the excessive PBQ covered the surface of BCO‐NCs and blocked the interfacial interaction between the catalyst and pollutant in water, resulting in the decrease of the system's removal performance for CIP.

Surprisingly, BCO‐NCs self‐purification system has ECs removal performance and simultaneous in situ H_2_O_2_ generation performance. As shown in Figure 2c, a significant H_2_O_2_ signal was detected in the suspension of BCO‐NCs regardless of the presence of CIP, which exhibited a sawtooth variation regarding the reaction time. Differently, the production of H_2_O_2_ was higher in the presence of CIP in the suspension compared with the absence of CIP, which was obviously closely related to the presence of CIP in the suspension. This phenomenon indicates that the removal of CIP and the production of H_2_O_2_ occurred simultaneously during the reaction.

The cleavage intermediates of CIP during the reaction were revealed by LC‐MS measurements (Figure 2d). CIP was detected at m/z = 331.9. After 60 min of reaction in the BCO‐NCs self‐purification system, a series of new fragments were detected, including large fragments at m/z = 364.7 and 315.8 (Figure S4, Supporting Information), which were the ring‐opening surface cleavage products, large fragments at m/z = 301.3, which were the hydroxylated products. Similarly, small molecular fragments at m/z = 245.9, 202.1, 157.9, 126.8, and 129.9 were also detected. In addition, the changes in F^−^ and NO_3_^−^ concentrations during the reaction were detected using ion chromatography, as shown in Figure 2e, both anion concentrations gradually increased as the reaction proceeded. These phenomena indicate that in the BCO‐NCs self‐purification system, CIP was decomposed into small molecules of carboxylic acids, alcohols, CO_2_, H_2_O, F^−^, and NO_3_^−^ within 60 min.

**Figure 3:**
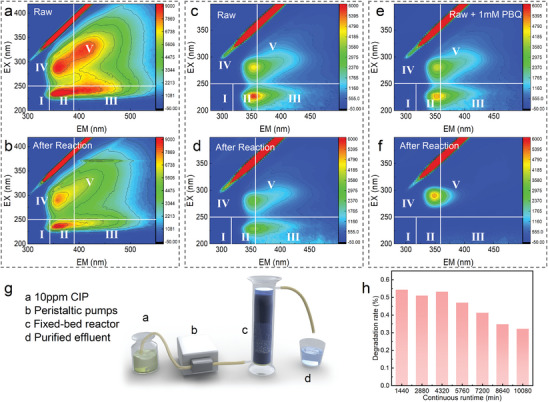
a) Fluorescence EEMs spectrum of raw kitchen wastewater; b) kitchen wastewater after 60 min of BCO‐NCs self‐purification system treatment. c) Fluorescence EEMs spectrum of raw pharmaceutical wastewater; d) pharmaceutical wastewater after 60 min of BCO‐NCs self‐purification system treatment. e) Fluorescence EEMs spectrum of raw pharmaceutical wastewater contained PBQ; f) pharmaceutical wastewater contained PBQ after 60 min of BCO‐NCs self‐purification system treatment. g) Schematic illustration of the fixed‐bed reactor (Hydraulic retention time = 60 min, under natural conditions). h) Degradation stability of CIP by BCO‐NCs self‐purification system in fixed‐bed reactor.

The purification effect on the actual wastewater is an important indicator to evaluate the practicality of the catalyst. Different types of actual wastewater were taken to evaluate the practicality of BCO‐NCs. Then, 3D excitation‐emission matrix (3D‐EEM) was used to detect fluorescent substances in the wastewater to evaluate the effectiveness of the BCO‐NCs self‐purification system for the treatment of this wastewater and classified into five regions based on previous research^[^ ^37^ ^]^ (regions I and II: simple aromatic proteins such as tyrosine; region III: small molecule organics such as fulvic acids; region IV: soluble microbial by‐products; and region V: acid‐like organics). The kitchen wastewater was taken from a shopping mall in southern China (113.32 E, 23.10 N). The 3D‐EEM spectrum of the kitchen wastewater before and after treatment is shown in **Figure** **3**a,b, the raw kitchen wastewater contained the characteristic peaks in regions II, III, IV, and V, indicating that the wastewater had a complex composition dominated by simple aromatic proteins and soluble microbial by‐products. After treatment by BCO‐NCs self‐purification system for 60 min, the characteristic peaks in regions III and V completely disappeared, and the intensity of the characteristic peaks in regions II and IV significantly decreased. Similarly, the BCO‐NCs self‐purification system showed excellent purification results for the dyeing wastewater (wastewater from a dyeing wastewater treatment plant in southern China, 116.28 E, 23.32 N). Significant removal of COD (ΔCOD = 88 mg L^−1^) was achieved during 90 min of purification, accompanied by a significant reduction of the 3D‐EEM signal (Figure S5, Supporting Information).

In addition, the pharmaceutical wastewater was taken from a pharmaceutical plant in southern China (113.27 E, 22.81 N). Figure 3c,d shows the 3D‐EEM spectrum before and after treatment. The raw pharmaceutical wastewater included only the characteristic peaks in regions II and IV, indicating the simple composition of the wastewater. Generally, pharmaceutical wastewater contains high concentrations of antibiotic‐like substances which inhibit microbial growth and result in a low biochemical compatibility. Similarly, after treatment by BCO‐NCs self‐purification system for 60 min, the intensity of the characteristic peaks in regions II and IV significantly decreased. After adding PBQ at a concentration of 1 mm to the raw pharmaceutical wastewater, the 3D‐EEM spectrum was not significantly changed, differently, the characteristic peak of region II in the treated 3D‐EEM spectrum completely disappeared (Figure 3e,f), which further confirmed the great promotion effect of PBQ on the water purification performance of this system.

The cycling stability of the catalyst is another important indicator to evaluate its practicality. A fixed‐bed reactor was built to evaluate the stability of the catalyst (Figure 3g,h). The reactor was operated with 300 mg of catalyst immobilized in quartz sand (to prevent catalyst loss), with a hydraulic retention time of 60 min, at ambient temperature and pressure without any additional energy/oxidant. No extra catalyst added during operation. About 35–55% (equivalent to 3.5–5.5 mg L^−1^) of the CIP could be stably removed in 10 080 min (168 operating cycles) under natural conditions.

The above phenomenon clearly verifies the high practicality of BCO‐NCs self‐purification system and the great potential in the purification of actual wastewater.

### 2.3 The Structure‐Activity Relationship of BCO‐NCs

**Figure 4:**
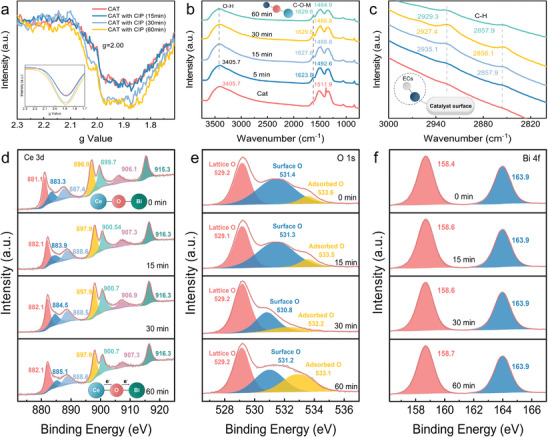
a) Solid EPR spectrum of BCO‐NCs self‐purification system at different reaction times (Inset shows the Gauss Amp fit curve). b,c) ATR‐FTIR spectrum of BCO‐NCs self‐purification system at different reaction times. d) Ce *3d* XPS orbit spectrum of BCO‐NCs at different reaction times. e) O *1s* XPS orbit spectrum of BCO‐NCs at different reaction times. f) Bi *4f* XPS orbit spectrum of BCO‐NCs at different reaction times. Reaction conditions: [Catalyst] = 1 g L^−1^, [CIP] = 100 mg L^−1^, [Temperature] = 35 °C.

To reveal the reason for the high practicality of BCO‐NCs under natural conditions, a series of characterizations were performed to investigate the structure‐activity relationship. Solid‐state EPR is frequently used to detect the signal of unpaired electrons in prepared samples. As shown in **Figure** **4**a, the intensity of unpaired electron signal on the surface of BCO‐NCs increased significantly as the reaction proceeded, indicating that interfacial electron transfer occurred on BCO‐NCs surface during the removal of CIP, and this transfer process was accelerated with time. Unexpectedly, contrary to the previous phenomenon,^[^ ^38^, ^39^, ^40^, ^41^, ^42^ ^]^ the solid EPR signal of BCO‐NCs was much weaker, which indicates that BCO‐NCs surface inherently had few unpaired electrons and weak surface polarity. Remarkably, H_2_O_2_ was not effectively activated by BCO‐NCs (Figure S6, Supporting Information), which might be attributed to the weak polarity of the surface of BCO‐NCs could not reach the oxidation–reduction potential (ORP) of H_2_O_2_ activation. H_2_O_2_ competes with CIP for adsorption in this case, resulting in a slight decrease in the CIP removal efficiency.

FTIR spectroscopy was used to detect changes in functional groups on the surface of BCO‐NCs during the reaction. As shown in Figure 4b, the broad absorption band centered at 3405.7 cm^−1^ was ascribed to the stretching vibration of OH [v(OH)] and the bending vibration of H‐O‐H [v(H‐O‐H)] in the water molecules on the catalyst surface.^[^ ^24^, ^43^ ^]^ As the reaction proceeds, the absorption band was significantly red‐shifted to 3415.3 cm^−1^, indicating that interactions between CIP and the metal species of the catalyst occurred,^[^ ^44^ ^]^ which may attribute to the complexation of the polar groups on the CIP surface with the metal species via hydrogen bonding or the aromatic structure on the CIP surface with the metal species via cation‐π interactions. In addition, a new absorption band at 1623.8 cm^−1^ appeared during the reaction, attributed to the stretching vibration of the C─O─M (metal species) [v(C‐O‐M)] on the catalyst surface,^[^ ^28^ ^]^ further confirmed the complexation of CIP with metal species on the surface of BCO‐NCs. As the reaction preceded, the absorption peak gradually blue‐shifted and increased in intensity, which was caused by the multiple decomposition products of CIP. Similarly, the absorption bands at 2935.1 and 2857.9 cm^−1^ appeared during the reaction (Figure 4c), attributed to the stretching vibration of C─H [v(C─H)].^[^ ^23^, ^45^ ^]^ The intensity of the C─H groups absorption band increased and then decreased, confirming the adsorption and cleavage processes of CIP on the catalyst surface.

XPS spectroscopy was used to investigate the change of elemental forms during the reaction. As shown in Figure 4d, Ce *3d* orbital XPS spectra was deconvoluted into seven characteristic peaks, including Ce (III) in the low wavenumber band and Ce (IV) in the high wavenumber band.^[^ ^46^ ^]^ The shift of the entire peak toward the high wavenumber band during the reaction was attributed to the decrease in electron density around Ce, indicating that the Ce site acted as an electron‐poor center in the reaction to remove ECs (Corresponding to the results of the XRD spectrum). O *1s* orbital XPS spectra was decomposed into three characteristic peaks (Figure 4e), including lattice O (Ce‐O‐Bi), surface O (O_2_, H_2_O), and adsorbed O (oxygen‐containing functional groups).^[^ ^47^ ^]^ The changes of surface O and adsorbed O during the reaction confirmed the formation of C─O─M bond bridges by the complexation of CIP with the catalyst (corresponding to FTIR spectra). Bi *4f* orbital XPS spectra were deconvoluted into two characteristic peaks (Figure 4f),^[^ ^48^ ^]^ which were attributed to Bi_2_O_3_ species, corresponding to the XRD results.

The above phenomena confirmed that the surface electron‐poor and electron‐rich centers (Ce as the electron‐poor center and Bi as the electron‐rich center) were constructed on the surface of BCO‐NCs. During the reaction, CIP exhibited an electron donating effect through cation‐π interaction with Ce center to achieve surface cleavage. Electrons were transferred to the Bi center through the Ce─O─Bi bond bridge due to the different electronegativity of Bi and Ce, which led to the decrease of electron density around Ce species. Dissolved oxygen (DO) in water might be activated by electrons at the Bi site as reactive oxygen species to further attack CIP, resulting in the degradation of CIP. PBQ, as an oxidizing substance, adsorbed at Bi site increased the polarity of BCO‐NCs and promoted the electron‐donating effect of CIP at the Ce site, leading to a significant increase in the removal efficiency.

### 2.4 Mechanism for BCO‐NCs to Utilize ECs Electrons

**Figure 5:**
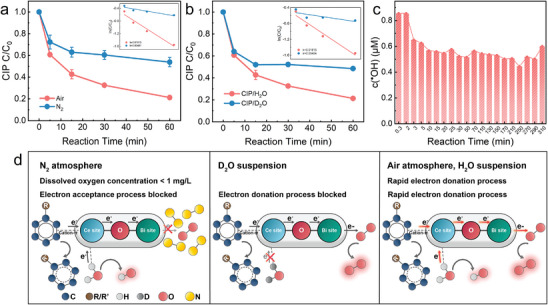
a) Comparison of CIP degradation in air and N_2_ atmospheres (the inset shows the pseudo‐first‐order kinetic rate plots). b) Comparison of CIP degradation in H_2_O and H_2_O_2_ suspensions (the inset shows the pseudo‐first‐order kinetic rate plots). c) Quantitative detection of ∙OH in aqueous suspensions using the terephthalic acid (TPA) probe method. d) Schematic illustration of the multi‐interface electron transfer mechanism in suspensions.

Investigating microscopic interfacial interactions is the key to revealing reaction mechanisms. To investigate the role of DO in the reaction process, CIP removal experiments were conducted in both air and nitrogen atmospheres. As shown in **Figure** **5**a, in N_2_ atmosphere (dissolved oxygen content in water less than 1 mg L^−1^), the removal rate of CIP by BCO‐NCs self‐purification system was only 1/4 of that in air atmosphere, indicating that DO could play a positive role in the degradation of CIP, corresponding to the promotion of CIP degradation by PBQ in Figure 2a. Similarly, the removal efficiency of CIP in D_2_O suspensions was only 1/4 of the efficiency in H_2_O (Figure 5b), indicating that H_2_O was involved in the interfacial process of CIP removal and played an active role in promotion. In addition, the catalytic activity of BCO‐NCs was surprisingly better than that of the classical Bi/Ce‐based photocatalytic system (Table S1, Supporting Information) although it was a self‐purification system without any external energy inputs, which demonstrated the excellent performance of the dual‐reaction center catalysts with asymmetric surfaces.

According to the previous research,^[^ ^49^ ^]^ during the degradation of pollutants, hydroxyl radicals (∙OH) could be produced due to their surface cleavage and hydrolysis process. Therefore, the generated ∙OH was quantitatively analyzed in BCO‐NCs self‐purification system using the terephthalic acid (TPA) probe method (Figure S7a, Supporting Information). In addition, a standard curve was constructed to quantify the ∙OH produced during the reaction by using a series of known concentrations of 2‐hydroxyterephthalic acid (*h*TPA) (Figure S7b,c, Supporting Information). As shown in Figure 5c, the ∙OH concentration reached a maximum of ≈0.85 µm during the first 2 min. Then it decreased abruptly to ≈0.65 µm, and the concentration of ∙OH remained stable and fluctuated through 310 min, indicating that the BCO‐NCs self‐purification system was able to stabilize the activation of DO and H_2_O utilizing the electrons/energy of pollutants. The schematic illustration of the multi‐interface electron transfer mechanism in suspensions was constructed based on the above phenomena (Figure 5d). In the N_2_ atmosphere, the electron acceptance process of Interface interaction is blocked due to the extremely low concentration of DO, and the cleavage of ECs is inhibited; similarly, in D_2_O suspension, the electron donation process of Interface interaction is blocked, and the cleavage of ECs is inhibited; in addition, the electron transfer channels in the system are unobstructed as DO and H_2_O are widely distributed in the solution.

Based on the above experimental phenomena, the interfacial reaction mechanism of BCO‐NCs to CIP removal was summarized as the following equation:

(1) $$\mathrm{BCO}-\mathrm{NCs}\;+\;R\;\to \;\mathrm{BCO}-\mathrm{NCs}\cdots e^{-}\;+\;R^{•}$$

(2) $$O_{2}\;+\;\mathrm{BCO}-\mathrm{NCs}\cdots e^{-}+\;H^{+}⇌{\mathrm{HO}}_{2}^{\cdot }+\mathrm{BCO}-\mathrm{NCs}$$

(3) $${\mathrm{HO}}_{2}^{\cdot }+\;\mathrm{BCO}-\mathrm{NCs}\cdots e^{-}+\;H^{+}⇌H_{2}O_{2}+\mathrm{BCO}-\mathrm{NCs}$$

(4)

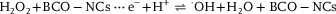



(5)

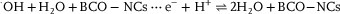




**Figure 6:**
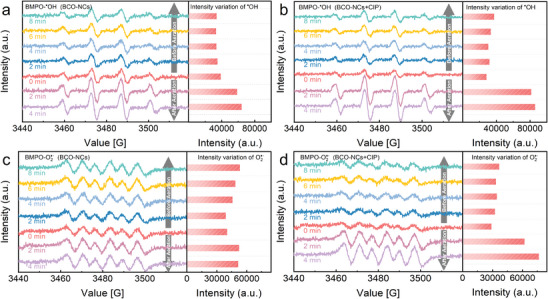
BMPO spin‐trapping EPR spectra of ∙OH in a) BCO‐NCs/H_2_O suspensions and b) BCO‐NCs/CIP/H_2_O suspensions before/after aeration with time (The bar graph on the right shows the variability of relative intensity). BMPO spin‐trapping EPR spectra of O_2_^∙−^ in c) BCO‐NCs/methanol suspensions and d) BCO‐NCs/CIP/methanol suspensions before/after aeration with time (The bar graph on the right shows the variability of relative intensity).

Meanwhile, EPR technology was also used to detect the radical signal generated during the reaction to further investigate this interfacial reaction process. A significant BMPO‐∙OH signal was detected in the BCO‐NCs system without CIP (**Figure** **6**a). Previous research^[^ ^28^ ^]^ suggested that the signal originated from the activation of DO by the catalytic system to produce ∙OH species, corresponding to Equation (2). The signal was progressively reduced with time, indicating that the weak surface polarity of the catalyst could not sustain activating DO to produce ∙OH (Equations (2), (3), (4)). Furthermore, combined with the phenomenon of D_2_O experiments, it was concluded that another origin of ∙OH in the BCO‐NCs system is probably obtained from the loss of one electron by H_2_O^[^ ^45^ ^]^ (Equation 5). Notably, the intensity of the signal increased significantly after introducing air into the system and increased 1.64 times within 4 min, suggesting that there were more electron acceptors as more oxygen was introduced into the system, thus leading to a multiplication of ∙OH signal. The ∙OH signal was enhanced after adding CIP to the system compared to the absence of CIP (Figure 6b), and fluctuated with time, indicating that CIP could act as an electron donor in the system, which allowed more DO to receive electrons to be reduced to ∙OH. Strikingly, the intensity of the signal increased significantly by 3.14 times after introducing air, which further confirmed the process that the CIP in the system acted as an electron donor, and electrons were transferred to DO through the Ce─O─Bi bond bridge on BCO‐NCs surface, and DO received electrons to be activated to ROS. This phenomenon was amplified with the increase of electron donor and acceptor concentrations, leading to a significant enhancement of the ∙OH signal.

The EPR signal of BMPO‐O_2_^∙−^ was observed in the suspension of BCO‐NCs without CIP (Figure 6c), indicating that single electrons around Bi species could be trapped by O_2_. This signal increased 1.32 times after 8 min, indicating that the surface polarity of BCO‐NCs could sustain activation of DO to O_2_^∙−^ (Equation 2). The increase in the intensity of this signal after introducing air further confirmed the activation of DO by BCO‐NCs, corresponding to the change in the BMPO‐OH signal. Differently, the signal of O_2_ was weakened after adding CIP (Figure 6d), indicating the involvement of O_2_ in the degradation chain reaction of CIP. Nevertheless, the signal intensity still increased with time, increasing to 2.6 times intensity within 4 min after introducing air, and the intensity was stronger than that in the absence of CIP. There was no ^1^O_2_ signal generated in the system (Figure S8, Supporting Information). This further confirmed that the surface cleavage of CIP was achieved by the electron‐donating effect in the system, simultaneously DO gained electrons to generate ROS for degrading CIP. DO was consumed in this process, and DO concentration increased with the air introduction, resulting in more DO gaining electrons to be reduced to ROS, and leading to a significant increase in ROS signal intensity.

Based on the above experimental results, ECs complexed on Ce sites on the surface of BCO‐NCs via cation‐π interaction during the reaction, and part of π electrons were transferred to the surface of BCO‐NCs. During this process, electron‐rich hydroxylation intermediates were formed with the cleavage of ECs (Figure 2d) and tended to be complex on Ce sites (the formation of C─O─M bond bridge. The increase in hydroxylation products resulted in the fluctuation of the ∙OH signal. Moreover, the electrons captured by BCO‐NCs from ECs were transferred to the electron‐rich center Bi site through the Ce─O─Bi bond bridge on the surface, reducing the dissolved oxygen adsorbed to O_2_∙− and H_2_O_2_. This research confirms that the BCO‐NCs self‐purification system is able to purify ECs in the aqueous environment and the actual wastewater without adding any external energy, which is completely different from conventional advanced oxidation processes.

## 3 Conclusion

Aiming to solve the global scientific challenge of exogenous energy consumption in water treatment technologies, the mechanism of H_2_O_2_ generation from dissolved oxygen in water by utilizing the inherent electron/energy activation of ECs without light/electricity or any external energy/oxidant input under natural conditions was realized. A high removal rate of ECs could be realized in the BCO‐NCs self‐purification system within a short time. Notably, ECs were completely removed within an extremely short time in the presence of abundant electron acceptors and the corresponding second‐order kinetic constants increased to 3.62 times. The mechanism shows that ECs complexed on Ce sites on the surface of BCO‐NCs via cation‐π interaction to exhibit electron‐donating effect, and the electrons obtained are transferred to Bi sites through Ce─O─Bi bond bridge to reduce the dissolved oxygen adsorbed there to O_2_∙− and H_2_O_2_. These findings would be a key advance in reducing energy consumption for environmental restoration and generating important chemical raw materials.

## 4 Experimental Section

### Preparation of Catalysts

BiCeO‐nanocubes (BCO‐NCs) were synthesized using a typical hydrothermal method. First, 4 mmol Ce(NO_3_)_3_·6H_2_O and 4 mmol Bi(NO_3_)_3_·5H_2_O were dissolved in 10 mL of dilute nitric acid and ultrasonicated for 30 min until the solution was clarified. Subsequently, 20 g of NaOH and 70 mL of deionized water were added to the solution and stirred continuously for 30 min to form a suspension. Finally, the suspension was transferred to an autoclave with a Teflon liner and maintained at a set temperature for 20 h. The metal species were embedded with each other in the hydrothermal environment and eventually formed BiCeO‐nanocubes (BCO‐NCs). As a reference, CeO was prepared without the addition of Bi(NO_3_)_3_·5H_2_O; BiO was prepared without the addition of Ce(NO_3_)_3_·6H_2_O.

### Characterization Techniques

The surface morphology and elemental composition information for the catalysts were detected by a field emission scanning electron microscope (FE‐SEM) (JSM‐6700F, JEOL Co., Japan) equipped with an energy dispersive X‐ray (EDX) detector system. High‐resolution transmission electron microscopy (HR‐TEM) (JEM‐2100, JEOL Co., Japan) was performed to further analyze the morphology and crystal structures of the catalysts. The X‐ray powder diffraction (XRD) patterns of the catalysts were recorded on a Philips X'Pert PRO SUPER diffractometer. Surface chemical information for the samples was collected via the X‐ray photoelectron spectroscopy (XPS) (VG Multilab 2000, Thermo Electron Co., America) using monochromatic Al Kα radiation (225 W, 15 mA, 15 kV) and low‐energy electron flooding for charge compensation. A Fourier transform infrared spectrometer (FTIR, Nicolet Is10) was used to investigate the surface functional groups of the catalyst, and the reaction process was observed through the ATR‐FTIR (TENSOR II+ Hyperion 2000, Bruker). Electron paramagnetic resonance (EPR, Bruker model A300‐10/12) was used to detect the unpaired electrons on the catalyst and reactive oxygen species (ROS).

### Experimental Procedure

Emerging contaminants ciprofloxacin (CIP) was chosen as the target contaminant due to the non‐biodegradation and potential toxicity. The concentration of pollutants was 10 mg L^−1^, and the catalyst dosage was 1.0 g L^−1^. Typically, 0.05 g of BCO‐NCs was added into a 50 mL solution containing CIP and stirred continuously under 35 °C for reaction. At given time intervals, 1 mL of solution was sampled and filtered through a Millipore filter (pore size 0.22 µm) and then analyzed through high‐performance liquid chromatography (HPLC Agilent 1260 Infinity HPLC (Agilent Co.) equipped with a UV detector and a Poroshell 120 EC‐C18 column (100 × 4.6 mm, 2.7 µm)). 1260–6460 LC/MS (Agilent) was used to analyze the generated intermediate products in CIP oxidation. Two‐time parallel experiments were conducted to calculate error bars.

A Fixed bed reactor containing BCO‐NCs was constructed for the reusability and stability tests. A peristaltic pump was used to pump 10 mg L^−1^ CIP solution into the reactor, and the water inlet mode of the reactor was at the bottom inlet and top outlet. The residence time was maintained at 60 min, and no additional oxidant was added during the reaction.

## Conflict of Interest

The authors declare no conflict of interest.

## Supporting information

Supporting Information

## Data Availability

The data that support the findings of this study are available from the corresponding author upon reasonable request.
