# Supplementary material for: Simultaneous Emerging Contaminant Removal and H2O2 Generation Through Electron Transfer Carrier Effect of Bi─O─Ce Bond Bridge Without External Energy Consumption
Source: Adv Sci (Weinh). 2024 Jun 3;11(29):2308519. doi: 10.1002/advs.202308519 (PMC11304260; doi:10.1002/advs.202308519)
Supplement: Supplementary file 1 — Supporting Information [file ADVS-11-2308519-s001.pdf]

ADVANCED SCIENCE
Open Access

Supporting Information

for *Adv. Sci.*, DOI 10.1002/advs.202308519

Simultaneous Emerging Contaminant Removal and H_2O_2 Generation Through Electron Transfer Carrier Effect of Bi—O—Ce Bond Bridge Without External Energy Consumption

*Yingtao Sun, Xuanying Cai, Yufeng Lai, Chun Hu and Lai Lyu**

Simultaneous Emerging Contaminant removal and H_2O_2 generation through electron transfer carrier effect of Bi-O-Ce bond bridge without external energy consumption

Yingtao Sun[1], Xuanying Cai[1], Yufeng Lai[1], Chun Hu[1], Lai Lyu[*1, 2]

[1]Key Laboratory for Water Quality and Conservation of the Pearl River Delta, Ministry of Education, Institute of Environmental Research at Greater Bay, Guangzhou University, Guangzhou 510006, China.

[2]Institute of Rural Revitalization, Guangzhou University, Guangzhou 510006, China.

E-mail: **Lai Lyu:** lyulai@gzhu.edu.cn.

Supplementary Experimental Section

Chemicals and Reagents. H_2O_2 (30% w/w) was purchased from General Reagent Co., China. ciprofloxacin (CIP) was purchased from Aladdin Co., China. Peroxidase from horseradish (POD) was purchased from Sigma Ltd. N, N-diethyl-p-phenylenediamine sulfate (DPD), Cerium nitrate ($Ce(NO_3)_3 \cdot 6H_2O$), Bismuth nitrate ($Bi(NO_3)_3 \cdot 5H_2O$) Terephthalic acid (TPA), 2-Hydroxy terephthalic acid (*h*TPA), 2,2,6,6-Tetramethyl-4-Piperidinol (TEMP), *p*-benzoquinone (PBQ) and $NH_3 \cdot H_2O$ (37% w/w)were purchased from Adamas Reagent Co., Ltd. 5-tert-butoxycarbonyl-5-methyl-1-pyrroline-N-oxide (BMPO) was purchased from Dojindo Molecular Technologies Inc., Japan. All of the other chemicals were of analytical grade. All water involved in the experiment was deionized water purified by EPED (Water Purifier Co., China).

HPLC Measurements. All of the pollutants were analyzed using a 1200 series HPLC (Agilent, U.S.A.) equipped with a UV detector and a ZORBAX Eclipse XDB-C_{18} column (4.6×150 mm, 5 μm). The mobile phase consisted of a 70/30 v/v mixture of methanol/water or 60/40 v/v mixture of acetonitrile/water at a flow rate of 1 mL min^{-1}.

HPLC-MS Analysis. High Performance Liquid Chromatography-Mass Spectrometry (HPLC-MS) analysis was conducted in auto full-scan mode (MS). A mixture of (A) water (0.25% formic acid) /(B) methanol was used as mobile phase. The column temperature was set at 40 ºC. Mobile phase procedure followed a gradient elution: 20% B were kept for first 1 min, and then increase to 98% B in 10 min. The flow rate was set at 0.3 mL min^{-1}, and 10 μL of the sample was injected. Mass spectral analysis was conducted in positive mode over a mass range of 50~500 m/z. The cone voltage was 20 V and desolvation temperature was 350 ºC.

3D-EEM fluorescence measurements. In general, 0.05 g of the catalyst powder was first

mixed with 50 mL of the actual wastewater (35 °C) in an appropriate volume glass beaker. The suspension was stirred throughout the experiment. At certain intervals, 3 mL reaction suspension was collected with a syringe and filtered with a filter (0.45 μm) for follow-up analysis.

Three-dimension excitation emission matrix (3D-EEM) fluorescence spectra of various samples were obtained on an F-7000 spectrometer (HITACHI) with a xenon excitation source, and slits were set to 5 nm for both excitation and emission. The excitation wavelengths were incremented from 200 to 450 nm in 5-nm steps; for each excitation wavelength, the emission was detected from 300 to 550 nm in 5-nm steps.

FTIR. The attenuated total reflection Fourier transform infrared spectroscopy (ATR-FTIR) was tested with a TENSOR FTIR spectrophotometer (Bruker Scientific Inc.) with a single ATR accessory. To prepare an ATR sample, 1 g L^{-1} catalyst was added to a 100 ppm CIP aqueous solution. The suspension was stirred at room temperature for approximately 30 min to establish adsorption/desorption equilibrium between the pollutant and the catalyst. Then the suspension was collected followed by filtration, the solid particles were collected and dried at approximately 50 °C to form the powder samples.

The FTIR spectra of these powder samples and fresh samples supported on KBr pellets at a fixed sample amount (1 wt%) were recorded on a Nicolet 8700 FTIR spectrophotometer (Thermo Fisher Scientific Inc., USA).

Raman and *in situ* EPR measurements. *in situ* Raman spectra for various catalyst were tested with an HR Evolution Raman spectrophotometer (HORIBA Scientific Inc.). The catalyst was placed directly into the reaction cell and scanned from 200 to 2000 cm^{-1} at a resolution of 1 cm-1 for 60 s with 40 mW 532 nm laser light irradiation.

For the EPR spectra measurement, BMPO/TEMP-trapped EPR signals were detected in different air-saturated methanol/aqueous dispersions of the corresponding samples using a Bruker A300-10/12 EPR spectrometer at room temperature (25°–30°). The center field is

3500 G, sweep width is 100 G, modulation frequency is 100 kHz. To detect $^{\bullet}OH$, 0.01 g of the prepared powder sample was added to 500 µL of water. Then, 100 µL of the above suspension, 20 µL of BMPO (250 mM) was mixed thoroughly and then left to stand for 1 min before being drawn into a capillary for detection. To detect $HO_2^{\bullet}/O_2^{\bullet-}$, the steps were the same as above except that water was replaced with methanol. To detect $^1O_2$, the steps were the same as that of detecting $^{\bullet}OH$ except that BMPO was replaced with TEMP.

Quantification of $^{\bullet}$OH by the TPA probe method. The generation of $^{\bullet}OH$ in the BCO-NCs aqueous dispersion was quantitatively measured using the terephthalic acid (TPA) probe method, in which the trapping of $^{\bullet}OH$ by TPA could generate the strongly fluorescent 2-hydroxyl-terephthalic acid (2-HTPA). The TPA solution was prepared with 4 mM of TPA and 12 mM of NaOH mixture. In a typical procedure, 50 mL of the TPA solution and 0.05 g of the catalyst powder were placed in a beaker. The pH value was adjusted to 6-6.5 using the aqueous hydrochloric acid solution. Then, continuous magnetic stirring at 35 °C throughout the experiment. At given time intervals, 3 mL aliquots were collected and filtered through a Millipore filter (pore size 0.45 µm) to detect the 2-HTPA ($^{\bullet}OH$) production based on the fluorescence intensity. The fluorescence intensity of the produced 2-HTPA was detected by a CARY ECLIPSE fluorescence spectrophotometer. The excitation and emission wavelengths of the detector were set at 310 and 425 nm, respectively.

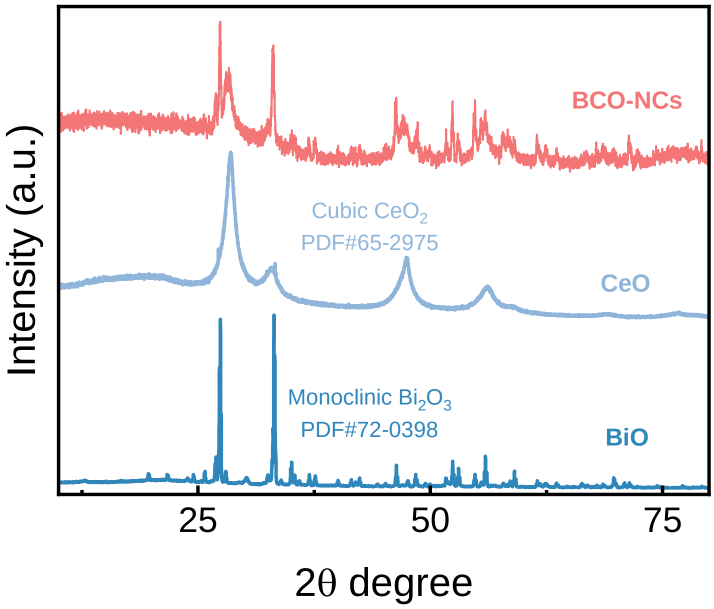

Figure S1. XRD patterns of BCO-NCs, CeO and BiO.

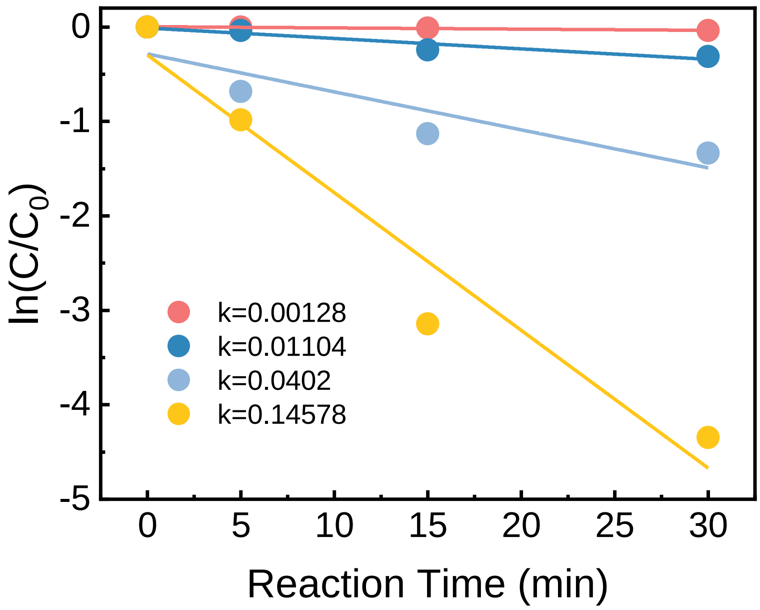

Figure S2. Second-order dynamical curves corresponding to **Figure 2a**. Reaction conditions: [natural initial pH] ~7.0, [catalyst] = 1 g L^{-1}, [temperature] = 35 °C.

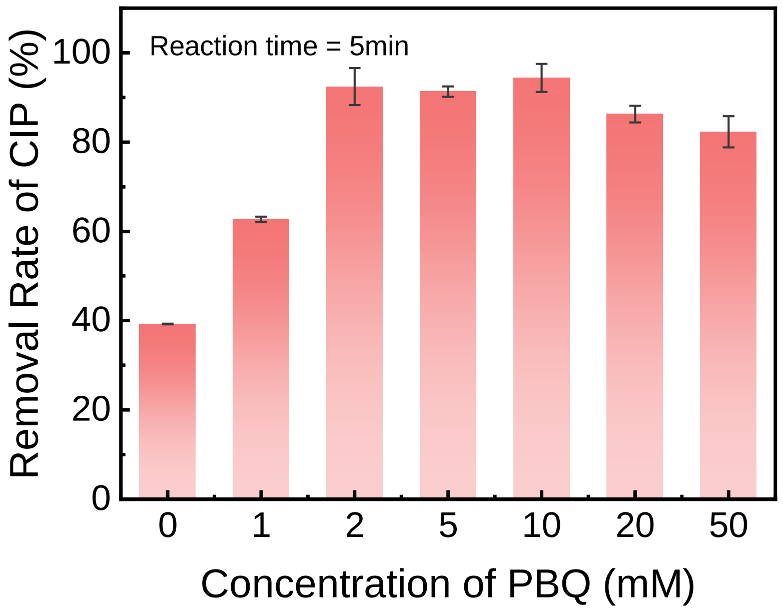

Figure S3. The removal rate of CIP at 5 min by adding different concentrations of PBQ.

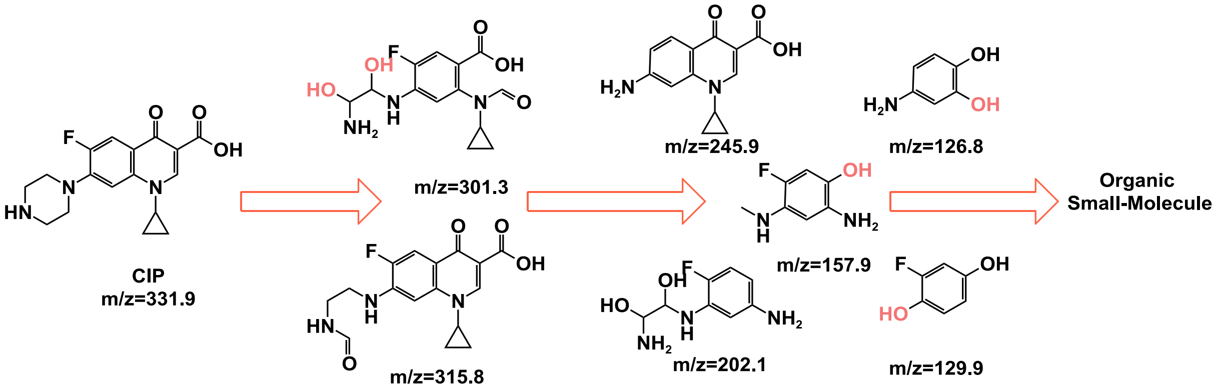

Figure S4. The proposed degradation pathways in the BCO-NCs system.

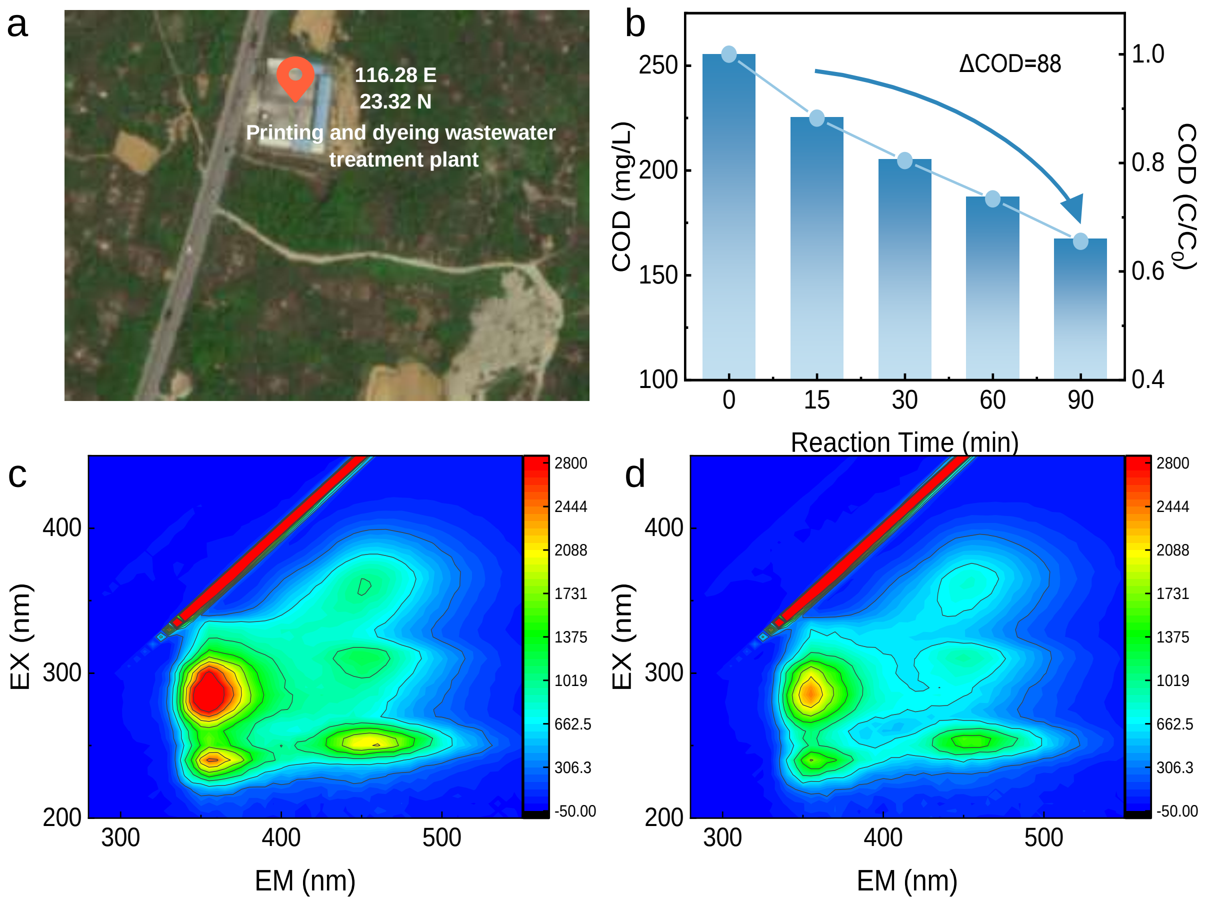

Figure S5. a Satellite map of printing and dyeing wastewater sampling sites. **b** Changes in COD of printing and dyeing wastewater during the purification process. **c** Fluorescence EEMs spectrum of raw printing and dyeing wastewater; **d** Printing and dyeing wastewater after 60 min of BCO-NCs self-purification system treatment.

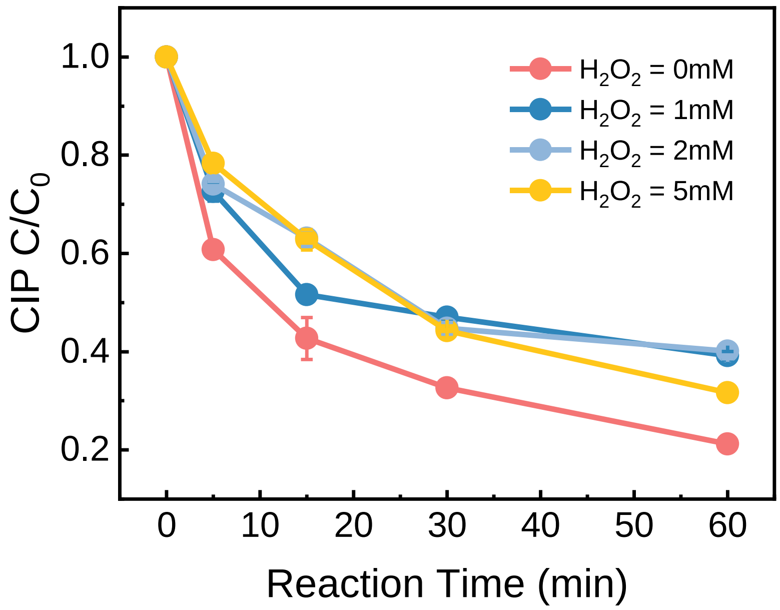

Figure S6. Effect of different concentrations of H_2O_2 on CIP removal.

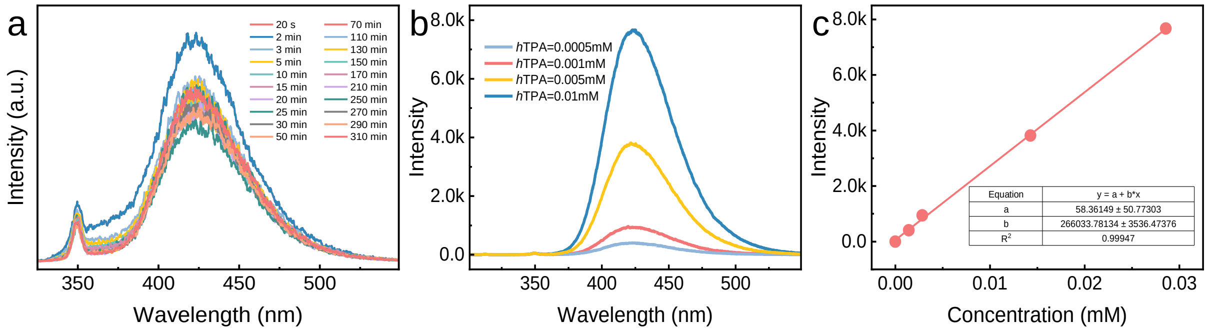

Figure S7. a Fluorescence spectral changes observed during the self-purification of BCO-NCs in terephthalic acid solution. (Excitation wavelength is 310 nm). **b, c** •OH concentration standard curve constructed using a series of known concentrations of 2-hydroxyterephthalic acid (*h*TPA).

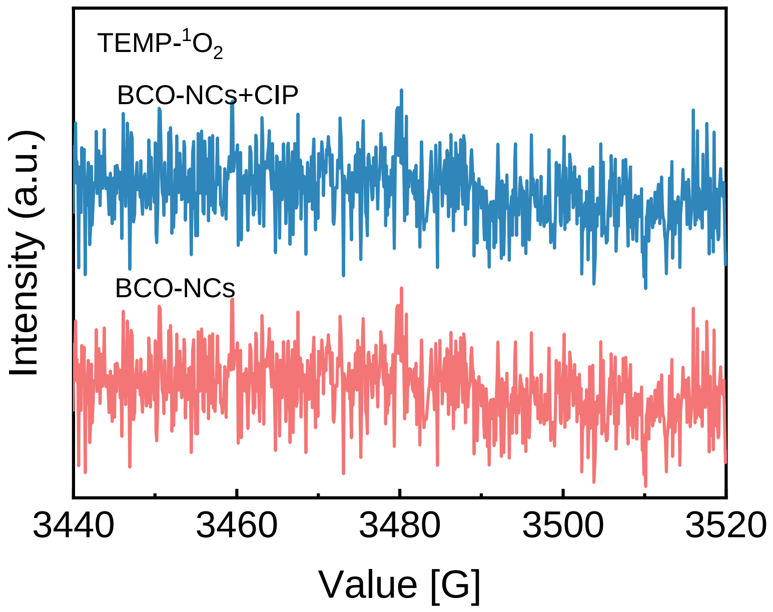

Figure S8. TEMP spin-trapping EPR spectra for $^1O_2$ in BCO-NCs suspensions with/without pollutants.

Table S1. Comparison of the catalytic performance with the previous reported catalysts.

Catalyst ($g\ L^{-1}$)	Pollutant ($mg\ L^{-1}$)	Energy input	Mechanism	k (min^{-1})
BCO-NCs (1)	CIP (10)	--	O_2 activation	0.018
3BCCN[1] (0.2)	SMX (10)	Xenon lamp 35 W	photocatalytic	0.004
2Ce-Bi/Bi2Ox[2] (1)	Lignin (100)	LED lamp (5 W, 80 mW/cm^2)	photocatalytic	0.014
3Ce-Bi/Bi2Ox[2] (1)	Lignin (100)	LED lamp (5 W, 80 mW/cm^2)	photocatalytic	0.008
5Ce-Bi/Bi2Ox[2] (1)	Lignin (100)	LED lamp (5 W, 80 mW/cm^2)	photocatalytic	0.006
$Ce_{0.2}$-BiOBr[3] (0.25)	BPA (10)	Xenon lamp (500W)	photocatalytic	0.009
$Ce_{0.1}$-BiOBr[3] (0.25)	BPA (10)	Xenon lamp (500W)	photocatalytic	0.01
Bi-PMOS[4] (0.33)	MB (20, pH=2)	Tungsten bulb (200 W)	photocatalytic	0.021
Ce-PMOS[4] (0.33)	MB (20, pH=2)	Tungsten bulb (200 W)	photocatalytic	0.008
Bi/Si-4[5] (2)	MB (10)	Xenon lamp (500W, 35 mW/cm^2)	photocatalytic	~0.004
Bi_2O_3: Zn[6] (2)	MB (1)	Sunlight	photocatalytic	0.021

References

1. Zhang, J.; Li, Y.; Gong, Y.; Zhu, C.; Zhang, L.; Tang, H.; He, W.; Wang, B., Bi(Ⅲ) and Ce(Ⅳ) functionalized carbon nitride photocatalyst for antibiotic degradation: Synthesis, toxicity, and mechanism investigations. *Chemosphere* **2023,** *333,* 138888.
2. Zhou, L.; Xie, M.; Rao, C.; Su, H.; Pang, Y.; Lou, H.; Yang, D.; Qiu, X., Oxygen vacancies induced by Ce^{3+}/Ce^{4+} doping mediate the formation of $Bi^0/Bi_2O_3/Bi_2O_{2.75}$ nanosheets for visible light-driven photocatalytic degradation of lignin. *Chem. Eng. J.* **2023,** 471.
3. Zeng, Q.; Wang, C.-Y.; Xu, B.-X.; Han, J.; Fang, X.; Zhu, G., Electron-level mechanistic insights into Ce doping for enhanced efficiency degradation of bisphenol a under visible light irradiation. *Nanomaterials* **2022,** *12*, (8), 1382.
4. Shahzad, K.; Imran Khan, M.; Elboughdiri, N.; Ghernaout, D.; ur Rehman, A., Energizing periodic mesoporous organosilica (PMOS) with bismuth and cerium for photo-degrading methylene blue and methyl orange in water. *Water Environ. Res.* **2021,** *93*, (7), 1116-1125.
5. Lu, H.; Hao, Q.; Chen, T.; Zhang, L.; Chen, D.; Ma, C.; Yao, W.; Zhu, Y., A high-performance Bi_2O_3/Bi_2SiO_5 p-n heterojunction photocatalyst induced by phase transition of Bi_2O_3. *Appl. Catal. B.* **2018,** *237*, 59-67.
6. Viruthagiri, G.; P, K.; Shanmugam, N., Photocatalytic rendition of Zn^{2+}-doped Bi_2O_3 nanoparticles. *Photonics Nanostruct* **2018,** *32*, 35-41.
